# Biomarkers of Extracellular Matrix Fragments in Patients with Psoriasis

**DOI:** 10.3390/ijms26010261

**Published:** 2024-12-30

**Authors:** Mila Broby Johansen, Signe Holm Nielsen, Helena Port, Tanja Todberg, Marianne Bengtson Løvendorf, Lone Skov

**Affiliations:** 1 Department of Dermatology and Allergy, Copenhagen University Hospital—Herlev and Gentofte, 2900 Hellerup, Denmark; tanja.todberg.01@regionh.dk (T.T.); marianne.bengtson.loevendorf@regionh.dk (M.B.L.); lone.skov.02@regionh.dk (L.S.); 2 Department of Clinical Medicine, University of Copenhagen, 2200 Copenhagen N, Denmark; 3 Nordic Bioscience, 2730 Herlev, Denmark; shn@nordicbio.com (S.H.N.); hep@nordicbio.com (H.P.); 4 The Leo Foundation Skin Immunology Research Center, Department of Immunology and Microbiology, Faculty of Health and Medical Sciences, University of Copenhagen, 2200 Copenhagen N, Denmark

**Keywords:** autoimmune skin disease, skin pathology, ECM, biomarkers, molecular pathology, treatment response

## Abstract

Blood-based extracellular matrix (ECM) fragments have been identified as potential pharmacologic biomarkers in spondyloarthritis and diagnostic biomarkers in psoriatic arthritis and psoriasis vulgaris. This study aimed to explore whether ECM fragments can differentiate patients with psoriasis from healthy controls (HC) and determine their potential as biomarkers for response to treatment in psoriasis. The study population included 59 patients with moderate to severe psoriasis, not receiving systemic anti-psoriatic treatment at inclusion, and 52 HC matched by age, sex, and BMI. An EDTA plasma sample was taken from all subjects at inclusion. Nine patients with psoriasis who initiated treatment with adalimumab after inclusion and responded successfully had an additional EDTA plasma sample taken after three to six months. Twelve ECM fragments were measured using validated ELISAs and Immunodiagnostic Systems automated chemiluminescent assays. C4M, indicating collagen IV degradation, PRO-C3, indicating tissue fibrosis, and PRO-C4, indicating epidermal basement membrane turnover showed significantly elevated levels in psoriasis patients compared with HC (*p* = 0.005, *p* = 0.016, and *p* = 0.018, respectively). Despite successful treatment, adalimumab did not alter C4M, PRO-C3, or PRO-C4 levels. In conclusion, compared with controls, C4M, PRO-C3, and PRO-C4 were elevated in psoriasispatients, but treatment did not modulate these fragments.

## 1. Introduction

Psoriasis (PSO) is a multifactorial chronic inflammatory skin disease [1]. The pathogenesis of psoriasis involves a combination of the innate and adaptive immune systems and is mainly driven by the Th1 and Th17 T-cells/IL-23 axis. Around 20% of patients with psoriasis develop psoriatic arthritis (PsA) [2,3]. Blood-based biomarkers may be an important supplement in the clinic to improve the monitoring of the patients. In PSO, only a limited number of biomarkers are available to predict disease progression, assess the risk of comorbidities, and evaluate response to systemic treatment. HLA-C*06:02 is one of the only more well studied biomarkers that predicts the response to ustekinumab, an IL-12/23 inhibitor [4,5]. Nevertheless, additional biomarkers are required.

The extracellular matrix (ECM) is a non-cellular network present in all tissues and organs, playing a vital role in maintaining structural integrity. In healthy skin, the ECM undergoes continuous regeneration, a process regulated by various ECM fragments to maintain balance in the skin [6]. Fibroblasts and keratinocytes are key contributors to tissue homeostasis and remodelling of the skin. Fibroblasts synthesise matrix-degrading enzymes such as matrix metalloproteases (MMPs) and are responsible for producing fibrillar collagens. The MMPs play a critical role in maintaining ECM stability by facilitating the cleavage of various ECM components, including elastin and collagens [7,8,9]. Collagens, the most abundant protein family in the ECM, are essential for maintaining the structural integrity and organisation of the skin’s different layers. Collagen types III and VI are present in the dermis, while collagen types IV, VII, XVII, and XXII are present in the epidermal basement membrane [7,10,11,12]. PRO-C4 is the most abundant in the basement membrane [13]. Collagens are synthesised from precursor procollagens (PRO-C) [13] and subsequently degraded by MMPs. Specific MMPs facilitate the release of distinct collagen fragments. For instance, C3M is a fragment released from collagen type III, C4M is a fragment released from collagen type IV, and C6M is a fragment released from collagen type VI. C4G is released from type IV collagen as a consequence of its degradation by the T-cell active protease, Granzyme B [14]. CPa9-HNE is a neo-epitope derived from an HNE-derived fragment of calprotectin, and one of its functions is to recruit leukocytes such as neutrophils. Neutrophils can release neutrophil extracellular trap formations (NETosis), which may be involved in the pathogenesis of psoriasis [15].

In PSO, the regenerative process of the ECM is disrupted. Research shows that certain proteins including heparanases, syndecans, elastin, tissue inhibitor metalloproteinase, laminin, and MMPs can alter ECM in psoriatic skin [8,9,16,17,18]. Among these, MMPs such as MMP-1, MMP-2, MMP-3, MMP-8, MMP-9, and MMP-12 play a role in the development of PSO. MMP-9 facilitates the release of the collagen type III fragment C3M, while other MMPs release the collagen type IV fragment C4M [9,17,19]. The inflammatory environment in PSO drives collagen degradation through the upregulation of MMP expression, leading to increased turnover and the release of ECM fragments into the bloodstream [6]. While the role of MMPs and collagen genes in PSO has been investigated [20,21], few studies have specifically focused on fragments derived from collagen formation and degradation, immune cell activity-related ECM fragments, or elastin degradation in PSO [7,12,17].

ECM fragments might be potential blood-based biomarkers for disease activity and treatment response in other diseases. For instance, in patients with axial spondyloarthritis, certain ECM fragments have been identified as biomarkers for response to TNF-α inhibitor treatment [22,23]. Additionally, blood-based ECM fragments have been explored in small studies as potential markers to distinguish between healthy controls and patients with psoriatic arthritis (PsA) or patients with PSO [12].

This study aimed to validate and expand upon these findings in a larger, well-defined cohort of patients with PSO. The central hypothesis was that ECM components contribute to psoriatic pathology. The specific objectives were to investigate whether ECM fragments can serve as biomarkers to distinguish patients with moderate to severe PSO from healthy controls (HC) and to evaluate the potential of ECM fragments as predictive biomarkers for treatment response in patients with PSO.

## 2. Results

### 2.1. ECM Fragment Levels in Patients with PSO Compared with HC

The baseline characteristics of patients and controls are shown in Table 1. The study cohort comprised 59 patients with PSO and 52 HC. The mean age was 47 years in the PSO group and 46 years in the HC group. Most of the participants were male, consisting of 33 individuals (56%) in the PSO group and 28 (54%) in the HC group. The median Psoriasis Area Severity Index (PASI) was 10.0 (8.0–36.8), and 15% (nine patients) had been diagnosed with PsA by a physician.

The analysed ECM fragments included: elastin degradation and neutrophil activity (ELP-3) [24], T-cell activity (C4G) [14], neutrophil activity (CPa9-HNE) [25], type III, IV, and VI collagen degradation (C3M [26], C4M [27], and C6M [28]), type III, VI, and VII collagen formation (PRO-C3 [29], PRO-C6 [30], and PRO-C7 [31]), and type IV, XVII, and XXII collagen turnover (PRO-C4 [32], PRO-C17 [33], and PRO-C22 [34]). Detailed information on each ECM fragment can be found in Table 2.

**Figure 1:**
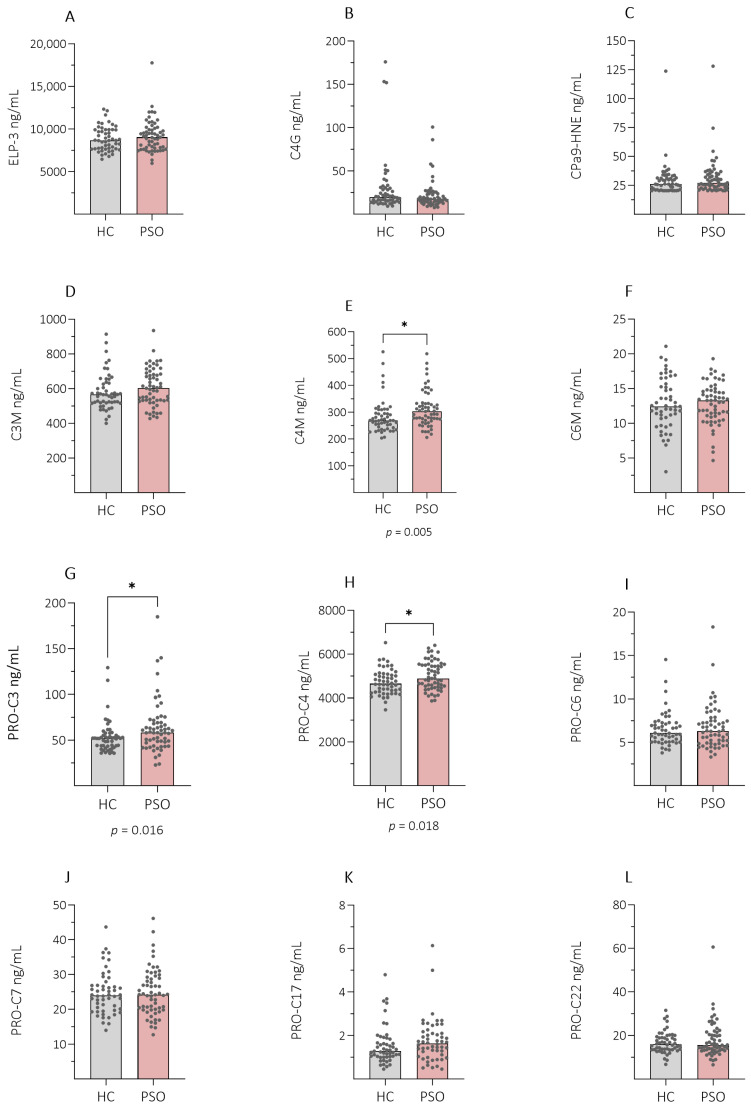
Comparison of ECM fragment levels between patients with PSO (PSO) and healthy controls (HC). Plasma levels of 12 ECM fragments were measured in healthy controls, (HC, n = 52), and patients with psoriasis (PSO, n = 59). We observed significantly elevated levels of C4M, assessing collagen IV degradation (*p* = 0.005), PRO-C3, assessing tissue fibrosis (*p* = 0.016), and PRO-C4, assessing epidermal basement membrane turnover (*p* = 0.018), in patients with PSO compared with HC (**E**,**G**,**H**). (**A**–**C**): Elastin degradation and neutrophil activity (ELP-3), T-cell activity (C4G), neutrophil activity (CPa9-HNE). (**D**–**F**): Catabolic ECM fragments of type III collagen (C3M), type IV collagen (C4M), type VI (C6M). (**G**–**L**): Anabolic ECM fragments of type III collagen (PRO-C3), type IV collagen (PRO-C4), type VI collagen (PRO-C6), type VII collagen (PRO-C7), type XVII (PRO-C17), and type XXII collagen (PRO-22). The significance threshold was set at *p* < 0.05 *, and data are presented as scatterplots with bars and lines at the median.

The differences in the levels of all 12 ECM fragments (ELP-3, C4G, CPa9-HNE, C3M, C4M, C6M, PRO-C3, PRO-C4, PRO-C6, PRO-C7, PRO-C17, PRO-C22) in patients with PSO compared with HC are presented in Figure 1 and Table S1. Significantly elevated levels of C4M, assessing collagen IV degradation (*p* = 0.005), PRO-C3, assessing tissue fibrosis (*p* = 0.016), and PRO-C4, assessing epidermal basement membrane turnover (*p* = 0.018), were observed in patients with PSO compared with HC (Figure 1E,G,H). No differences were observed in the remaining fragment levels between patients with PSO and HC (Table S1).

No significant difference was found in ECM fragment levels between patients with PSO without PsA (n = 50) and HC compared with patients with PSO with PsA (n = 9) (Figure S1).

### 2.2. ECM Fragment Levels in Patients with PSO with a Successful Treatment Response

To determine the potential of ECM fragments as biomarkers for response to treatment in patients with PSO, the three ECM fragments elevated in patients with PSO (C4M, PRO-C3, and PRO-C4), were selected to be measured after treatment in a selected group of nine good responders (PASI ≤ 2).

**Figure 2:**
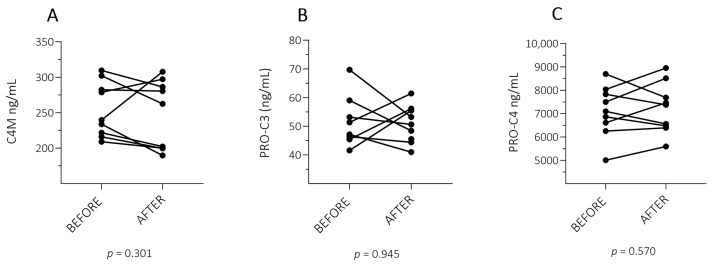
Levels of EMC fragments C4M, PRO-C3, and PRO-C4 before and after successful treatment response in patients with PSO. Plasma levels of three ECM fragments were measured at inclusion and 3–6 months after adalimumab treatment in patients with PSO with a successful treatment response (n = 9). No significant change was observed in C4M, PRO-C3, and PRO-C4 before and after treatment in the group with a successful response. (**A**): Catabolic ECM fragments of type IV collagen (C4M). (**B**,**C**): Anabolic ECM fragments of type III collagen (PRO-C3) and type IV collagen (PRO-C4). The significance threshold was set at *p* < 0.05.

The nine patients had a mean age of 36 years, the majority being males (56%) and with a median baseline PASI of 10.2 (8.1–21.4) and 0.0 (0.0–2.0) 3–6 months after biological treatment. One patient had PsA (Table 1). No significant change was observed in levels of C4M (*p* = 0.301), PRO-C3 (*p* = 0.945), and PRO-C4 (*p* = 0.570) when comparing before and after treatment values in the group with a successful treatment response (Figure 2).

## 3. Discussion 

This study measured a panel of ECM fragments to identify if ECM fragments showed any potential to differentiate patients with PSO from HC and as a secondary aim, if the selected ECM fragments were modulated during treatment. The results demonstrated a small but significant increase in C4M, PRO-C3, and PRO-C4 levels in patients with PSO compared to HC, but no change in the measured markers after treatment. Most of the degradation fragments measured in this study are released by MMP-9. In the literature, different MMPs have been shown to impact tissue remodelling in the psoriatic epidermis [7,9]. However, there are limited reports on elastin degradation, immune cell activity-related ECM fragments and ECM fragments derived from collagen formation and degradation in PSO (Table 2) [7,12,17].

Contrary to our findings, C4M and PRO-C4 were not elevated in a small study of patients with PSO; the authors found decreased levels of C3M in patients with PSO and patients with PsA compared with HC. Furthermore, they found elevated levels of PRO-C3 in both patients with PsA and patients with PSO. The latter finding with elevated PRO-C3 in patients with PSO is in line with the results of this study [12]. Another study by Holm Nielsen et al. showed elevated levels of PRO-C4, C4M, and PRO-C3 in patients with PsA compared with HC, but no difference in C3M was found [35]. C3M is a fragment of type III collagen. PRO-C3 is mainly found in connective tissue and primarily reflects type III collagen formation by fibrogenesis and healing. PRO-C3 peptides have often been used as a marker of liver fibrosis in patients with PSO treated with methotrexate; however, they were eventually replaced by fibrosis-4 (FIB-4) [36,37]. Measurement of PRO-C3 peptides has been used primarily in dermatology and it is well known that patients with active arthritis can have elevated levels [38]. Only nine patients in this study had PsA. In a small group, the differences in the ECM fragments were examined between patients with PSO without PsA, HC, and patients with PSO with PsA. However, no evidence of differences was found. Studies investigating early diffuse systemic sclerosis, where fibrosis is developed in the skin and internal organs, also observed elevated levels of PRO-C4 and PRO-C3 compared with HC [39,40]. Our findings support that increased fibrogenesis reflected by excessed type III collagen formation play a role in the pathogenesis of psoriasis [41,42]. The contrary findings of C4M, PRO-C4, and C3M in patients with PSO compared with HC indicate that the direct function of these fragments in patients with PSO remains unknown.

Previous studies have demonstrated the potential of ECM fragments to illustrate the pharmacologic effects of biological treatments in patients with spondyloarthritis and inflammatory bowel disease [22,23,43]. Port et al. has shown that C1M, C3M, C4M, C6M, CRP, PRO-C4, and CPa9-HNE levels declined after 12 weeks in patients with spondyloarthritis treated with adalimumab compared with placebo [23], but on the contrary, this was not confirmed in another study with patients with spondyloarthritis 22 weeks after adalimumab treatment [22]. C4M has been shown to predict treatment response in patients with inflammatory bowel disease [43]. However, in this small study with a subgroup of a limited number of patients with a successful treatment response, C4M, PRO-C3, and PRO-C4 levels were not modulated by a successful treatment with adalimumab (PASI ≤ 2). This was an explorative study that only consisted of nine patients and the results should be interpreted with caution.

Additionally, various factors and pathological processes in different tissues can lead to an imbalance of ECM fragments and their release into the bloodstream. These factors include lean body mass, liver fibrosis, alcohol binge drinking, pulmonary fibrosis, lung diseases, and other inflammatory diseases [6,43,44,45,46,47,48,49,50]. In addition, the immune-cell activity-derived fragment, CPa9-HNE, quantifies activated neutrophils and has been associated with patients affected by inflammatory bowel diseases and hidradenitis suppurativa [25,51]. Neutrophils also play an important role in psoriasis pathogeneses, especially in systemic inflammation, and are found in the dermis and epidermis and form Munro’s microabscesses [52]. Despite that, there were no changes in the immune-cell activity-derived fragments (CPa9-HNE and C4G) from patients with PSO compared with HC in this study. However, the group of patients with PSO had a low BMI, and individuals with diabetes, autoimmune diseases, cancer, or infections were excluded from the study [53]. This exclusion helps to minimise the risk of other disease-affected tissues influencing the results in this cohort. This complexity of different factors that can influence ECM fragments release into the blood makes it challenging to determine the exact cause of the differences in ECM fragments between patients with PSO and HC. These differences may be due to the skin pathology in psoriasis or other factors and affected tissues [54,55]. Therefore, we cannot determine if these ECM fragments measured in this study are specific for patients with PSO. The ECM fragments may be more specific for PsA as other studies have indicated.

### Strengths and Limitations

The strength of this study is the large cohort of patients with untreated moderate to severe psoriasis and with a carefully matched control group. Few studies have investigated the potential of ECM fragments as biomarkers to differentiate between patients with moderate to severe psoriasis and HC and their potential as biomarkers for response to treatment in patients with PSO. A limitation of this study is the relatively limited sample size of patients with PSO who had a successful treatment response and PsA. A longitudinal prospective study with a larger patient cohort is necessary to gain a more comprehensive understanding of the potential of ECM fragments, as a measure of anti-psoriatic treatment efficacy.

## 4. Materials and Methods

### 4.1. Study Design and Study Population

The study population comprised patients with PSO who were not receiving systemic anti-psoriatic treatment and HC matched by age, sex, and BMI. The population has previously been described [53,56]. All participants provided informed consent before their enrolment in the study. The study was approved by the ethics committees of the Capital Region of Denmark (H-18041455) on the 22 October 2018 and by the Danish Data Protection Agency (P-2020-747) on the 19 July 2023. The study was conducted according to the Helsinki Declaration. Inclusion criteria were individuals aged 18–74, residing in the capital region of Denmark, with a BMI < 35 kg/m^2^, baseline EDTA plasma blood samples at inclusion, a diagnosis of plaque psoriasis for more than six months, no systemic anti-psoriatic treatment for ≥3 months, and PASI ≥ 8 at inclusion. HC had the same inclusion criteria as patients with PSO excluding the psoriasis-specific criteria. Patients with PSO were recruited from the outpatient clinic of the Department of Dermatology and Allergy, Herlev and Gentofte Hospital, Copenhagen, Denmark. Nine patients with PSO who commenced adalimumab treatment after inclusion and exhibited a successful treatment response (defined as a PASI score ≤ 2 post-treatment) were selected for an explorative follow-up study, and a new EDTA plasma blood sample was collected after 3–6 months.

### 4.2. ECM Fragments Assessment in EDTA Plasma

Upon their collection, the EDTA plasma blood samples were stored at −80 °C until analysis. A panel of 12 ECM fragments was measured using validated ELISA (Enzyme-Linked Immunosorbent Assay) and Immunodiagnostic Systems (IDS) automated chemiluminescent assays, at Nordic Bioscience, Herlev, Denmark. The assays were previously developed and technically validated; detailed information on each ECM fragment can be found in Table 2. Sample measurements were performed in duplicates, and values were accepted when the standard curve had a coefficient of variance ≤ 10% and if at least three of the five control samples had a coefficient of variance ≤ 20%. Before measurements, technical assay parameters were validated according to Nordic Bioscience criteria. For more detailed information of the method, please refer to Supplementary Text S1.

### 4.3. Statistical Analysis

Baseline characteristics are presented as mean with standard deviation (SD) for normally distributed continuous variables and median with range (minimum to maximum) for non-normally distributed continuous variables. For categorical variables, numbers were used with frequencies expressed as percentages. Quantile–quantile plots, Gaussian normal distribution, and the D’Agostino–Pearson normality test were used to assess the data distribution. The Mann–Whitney U-test was used to analyse any differences in ECM fragment levels between independent groups: (1) patients with PSO and HC, (2) patients with PSO with PsA compared to HC, and (3) patients with PSO with PsA compared to patients with PSO without PsA. The response to treatment in patients with PSO with successful treatment response (PASI ≤ 2) was analysed using the Wilcoxon paired (signed-rank) test. Analysis and graphs were conducted in GraphPad Prism (version 10). A *p*-value below 0.05 was considered significant.

## 5. Conclusions

The results demonstrated that the ECM fragments C4M, PRO-C3, and PRO-C4 were slightly elevated in patients with PSO compared with HC, although their specific clinical role in psoriasis remains unclear. Treatment of psoriasis did not appear to modulate the levels of these fragments.

## Figures and Tables

**Table 1:** Baseline characteristics.

Variables	Patients with PSO (n = 59)	PSO After Treatment (n = 9)	Healthy Controls (n = 52)
Age, years, mean (SD)	47 (±16)	36 (±12)	46 (±14)
Men, n (%)	33 (56%)	5 (56%)	28 (54%)
Age at diagnosis, years, mean (SD)	24 (±16)	17 (±10)	-
Family history, n (%)			
Yes	33 (56%)	5 (56%)	-
No	23 (39%)	2 (22%)	-
Unknown	4 (7%)	2 (22%)	-
PASI all patients, median (min-max)	10.0 (8.0–36.8)	-	-
PASI before treatment, median (min-max)	-	10.2 (8.1–21.4)	-
PASI after treatment, median (min-max)	-	0.0 (0.0–2.0)	-
Psoriatic arthritis (PsA), n (%)	9 (15%)	1 (11%)	-
Age at diagnosis, years, median (min-max)	31 (16–42)	-	-

PSO: psoriasis, HC: Healthy controls, PsA: psoriasis arthritis, n: number, SD: standard deviation, %: percent, PASI: Psoriasis Area Severity Index, min: minimum, max: maximum. Means with standard deviation (SD) for normally distributed continuous variables and median with range (minimum to maximum) for non-normally distributed continuous variables. Number and frequency as percentages for categorical variables.

**Table 2:** Panel of 12 extracellular matrix (ECM) fragments measured in this study.

Abbreviation	Description	Function	Reference
ELP-3	Neo-epitope of proteinase-3 mediated degradation of elastin	Degradation of elastin	[24]
C4G	Fragment of type IV collagen released by granzyme-B	T-cell migration through the basement membrane by the degradation of type IV collagen	[14]
CPa9-HNE	Fragment of S100A9 (calprotectin) released by neutrophil elastase	Neutrophil activity and neutrophil extracellular trap formation (NETosis)	[25]
C3M	Fragment of type III collagen released by MMP-9	ECM degradation	[26]
C4M	Fragment of type IV collagen released by MMP (multiple)	Basement membrane degradation and ECM degradation	[27]
C6M	Fragment of type VIa1 collagen released by MMP-2/9	Interface matrix degradation and fibrogenesis	[28]
PRO-C3	Fragment of N-terminal type III collagen	Fibrogenesis and ECM formation	[29]
PRO-C4	Fragment of the internal 7S domain of type IV collagen	Basement membrane synthesis and ECM formation	[32]
PRO-C6	Fragment of C-terminal type VIa3 collagen (Endotrophin)	Pro-fibrotic signalling and ECM formation	[30]
PRO-C7	Fragment of C-terminal type VII collagen	ECM formation	[31]
PRO-C17	Fragment of C-terminal type XVII collagen	ECM formation and epithelial damage	[33]
PRO-C22	C-terminal of type XXII collagen, neoepitope-specific	Connective tissue formation	[34]

MMP: Metalloproteinase, ECM: Extracellular matrix, HNE: Human elastase, NET: Neutrophil extracellular traps.

## Data Availability

The data presented in this study are available on request from the corresponding author.

## Supplementary Materials

The following supporting information can be downloaded at: https://www.mdpi.com/article/10.3390/ijms26010261/s1.
