# Supplementary material for: Biomarkers of Extracellular Matrix Fragments in Patients with Psoriasis
Source: Int J Mol Sci. 2024 Dec 30;26(1):261. doi: 10.3390/ijms26010261 (PMC11720200; doi:10.3390/ijms26010261)
Supplement: Supplementary file 1 [file ijms-26-00261-s001.zip › ijms-3356930-supplementary.pdf]

Supplementary Table S1. ECM fragment levels in patients with psoriasis (PSO) compared with healthy controls (HC). The table shows *p*-values for comparing ECM fragment levels between patients with PSO and HC.

Fragments (ng/mL)	PSO (n = 59)	HC (n = 52)	*p*-value
	**Median (min-max)**	**Median (min-max)**	
ELP-3	9023.00 (5969.00-17758.00)	8652.00 (6437.00-12331.00)	0.432
C4G	17.55 (7.65-100.60)	19.83 (9.13-175.90)	0.212
CPa9-HNE	27.14 (20.36-128.00)	26.17 (20.36-123.70)	0.260
C3M	602.30 (427.60-934.60)	565.60 (399.80-914.30)	0.140
C4M	302.8 (206.00-518.10)	269.10 (203.60-525.50)	**0.005**
C6M	13.28 (4.63-19.29)	12.41 (3.01-21.08)	0.822
PRO-C3	58.19 (22.78-184.80)	51.94 (35.49-129.10)	**0.016**
PRO-C4	4889.00 (3874.00-6405.00)	4647.00 (3464.00-6523.00)	**0.018**
PRO-C6	6.27 (3.31-18.28)	6.07 (3.78-14.53)	0.943
PRO-C7	24.21 (12.68-46.12)	23.95 (19.95-43.67)	0.806
PRO-C17	1.63 (0.45-6.13)	1.28 (0.45-4.79)	0.100
PRO-C22	15.56 (6.56-60-54)	15.91 (6.73-31.49)	0.898

PSO: psoriasis, HC: healthy controls, ng: nanogram, mL: milliliter, N: number, min: minimum, max: maximum, ELP-3: Neo-epitope of proteinase-3 mediated degradation of elastin, C4G: A fragment of type IV collagen released by granzyme-B, CPa9-HNE: A fragment of S100A9 (calprotectin) released by neutrophil elastase, C3M: A fragment of type III collagen released by MMP, C4M: A fragment of type IV collagen released by MMP, C6M: A fragment of type VIa1 collagen released by MMP-2, PRO-C3: A fragment of N-terminal type III collagen, PRO-C4: A fragment of the internal 7S domain of type IV collagen, PRO-C6: A fragment of C-terminal type VIa3 collagen (Endotrophin), PRO-C7: A fragment of C-terminal type VII collagen, PRO-C17: A fragment of C-terminal type XVII collagen, PRO-C22: C-terminal of type XXII collagen, neoepitope specific. Statistical differences are analysed using Mann-Whitney U-test with a median (range from min-max). The significance threshold was set at $p < 0.05$, and significant results were highlighted in **bold**.

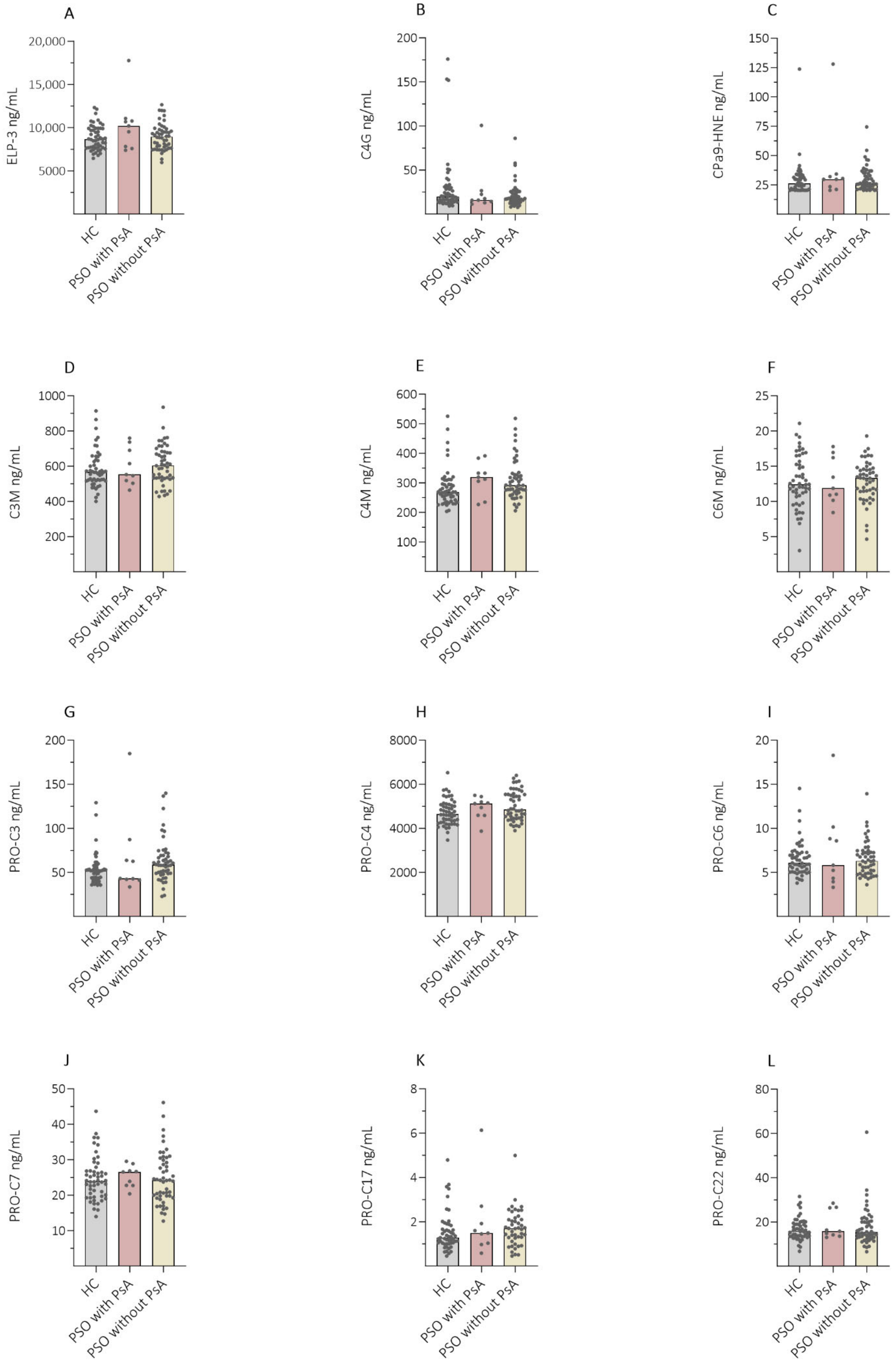
A
ELP-3 ng/mL
B
C4G ng/mL
C
CPa9-HNE ng/mL
D
C3M ng/mL
E
C4M ng/mL
F
C6M ng/mL
G
PRO-C3 ng/mL
H
PRO-C4 ng/mL
I
PRO-C6 ng/mL
J
PRO-C7 ng/mL
K
PRO-C17 ng/mL
L
PRO-C22 ng/mL
HC
PSO with PsA
PSO without PsA

Supplementary Figure S1. Comparison of ECM fragment levels between HC and patients with PSO with PsA, and between patients with PSO without PsA and patients with PSO with PsA. Plasma levels of 12 ECM fragments were measured in healthy controls, (HC, n = 52), patients with PSO with PsA (n = 9), and patients with PSO without PsA (n = 50). A comparison of HC and patients with PSO with PsA, and patients with PSO with PsA and patients with PSO without PsA showed no significant differences. A-C: Elastin degradation and neutrophil activity (ELP-3), T-cell activity (C4G), neutrophil activity (CPa9-HNE). D-F: Catabolic ECM fragments of type III collagen (C3M), type IV collagen (C4M), type VI (C6M). G-L: Anabolic ECM fragments of type III collagen (PRO-C3), type IV collagen (PRO-C4), type VI collagen (PRO-C6), type VII collagen (PRO-C7), type XVII (PRO-C17), and type XXII collagen (PRO-22). The significance threshold was set at $p < 0.05$, and data are presented as scatterplots with bars and lines at the median.

Supplementary Text S1: Detailed method description for the detection of ECM fragments.

Assays used either colorimetry or chemiluminescence as the detection method. Ninety-six well plates pre-coated with streptavidin were prepared by coating them with biotinylated peptides specific to each biomarker for 30 minutes at 20°C. Samples were diluted in assay buffer (PBS-BTB with NaCl, pH 7.4) and then incubated with horseradish peroxidase-conjugated target-specific monoclonal antibodies. Incubation was carried out either for 1 hour at 20°C or for 20 hours at 4°C, depending on the assay requirements, with shaking at 300 rpm. After each incubation step, the plates were washed using a washing buffer (25 mM TRIZMA, 50 mM NaCl, 0.036% Bronidox L5, 0.1% Tween-20) with a standardized ELISA plate washer (BioTek Instruments, Microplate Washer, ELx405 Select CW).

For colorimetric assays, 100 µl of tetramethylbenzidine (TMB) solution (Kem-En-Tec) was added to each well, and the plates were incubated at room temperature for 15 minutes while shaking at 300 rpm. The reaction was stopped by adding 1% H2SO4. Optical densities were measured at 450 and 650 nm using an ELISA reader (VersaMAX; Molecular Devices).

For chemiluminescence assays, 100 µl of BM Chemiluminescence ELISA Substrate (Merck, CAT no. 11582950001) was added per well, followed by incubation for 3 minutes at 20°C with shaking at 300 rpm. Light emission was detected using a fluorescence plate reader (Fluoroskan FL, Thermo Fisher) with a measurement setting of 1000 ms and no filter.

Finally, standard curves were generated using four-parameter logistic models to quantify assay results.
